# Differences in adhesion and protrusion properties correlate with differences in migration speed under EGF stimulation

**DOI:** 10.1186/2046-1682-5-8

**Published:** 2012-05-11

**Authors:** Yue Hou, Sarah Hedberg, Ian C Schneider

**Affiliations:** 1 Department of Chemical and Biological Engineering, Iowa State University, Iowa, USA; 2 Department of Genetics, Development and Cell Biology, Iowa State University, Iowa, USA; 3 Department of Biochemical Engineering, University College London, London, UK

## Abstract

**Background:**

Cell migration plays an essential role in many biological processes, such as cancer metastasis, wound healing and immune response. Cell migration is mediated through protrusion and focal adhesion (FA) assembly, maturation and disassembly. Epidermal growth factor (EGF) is known to enhance migration rate in many cell types; however it is not known how FA maturation, FA dynamics and protrusion dynamics are regulated during EGF-induced migration. Here we use total internal reflection fluorescence (TIRF) microscopy and image analysis to quantify FA properties and protrusion dynamics under different doses of EGF stimulation.

**Results:**

EGF was found to broaden the distribution of cell migration rates, generating more fast and slow cells. Furthermore, groups based on EGF stimulation condition or cell migration speed were marked by characteristic signatures. When data was binned based on EGF stimulation conditions, FA intensity and FA number per cell showed the largest difference among stimulation groups. FA intensity decreased with increasing EGF concentration and FA number per cell was highest under intermediate stimulation conditions. No difference in protrusion behavior was observed. However, when data was binned based on cell migration speed, FA intensity and not FA number per cell showed the largest difference among groups. FA intensity was lower for fast migrating cells. Additionally, waves of protrusion tended to correlate with fast migrating cells.

**Conclusions:**

Only a portion of the FA properties and protrusion dynamics that correlate with migration speed, correlate with EGF stimulation condition. Those that do not correlate with EGF stimulation condition constitute the most sensitive output for identifying why cells respond differently to EGF. The idea that EGF can both increase and decrease the migration speed of individual cells in a population has particular relevance to cancer metastasis where the microenvironment can select subpopulations based on some adhesion and protrusion characteristics, leading to a more invasive phenotype as would be seen if all cells responded like an “average” cell.

## Background

Cell migration plays an important role in tumor progression [1]. During invasion and metastasis, migration is driven by soluble extracellular cues like epidermal growth factor (EGF). EGF is a well-known chemoattractant [2-4]; however, uniform doses also stimulate chemokinetic responses. EGF’s control of cell motility originates from its regulation of adhesion and protrusion [5-7]. This occurs through altering adhesive attachments called focal adhesions (FAs) [8-10] as well as actin cytoskeleton organization.[8,11,12] The response to EGF at the level of cell migration is dose dependent, but there exists a range of maximal stimulation concentrations. Often migration saturates at 2–10 nM EGF [2,13,14], but some of the studies showed an inhibition of migration at EGF concentrations >2-10 nM [7,15]. This is in agreement with other work demonstrating that in certain contexts, EGF can inhibit migration [16,17]. Within each study there is wide diversity in migration behavior, even among cells observed during the same experiment [14]. Interestingly, the distribution in migration speed and persistence time appears to be dependent on EGF stimulation [18], suggesting that EGF controls not only the mean response, but also the amount of cell-to-cell variability. Cell-to-cell variability has been widely observed, and has drawn much attention due to its influence on physiology [19], pathology[20] and pharmacology [21]. Consequently, mathematical models [19,20] have been used to show that even small changes in the distribution of protein concentrations yield enhanced wound healing or metastasis due to the selection of an optimal subpopulation. When the subpopulation is defined based on migration speed, it will not only be beneficial to examine the distribution of protein concentrations, but also higher level characteristics like focal adhesion (FA) properties and cytoskeletal dynamics.

FAs are dynamic, macromolecular structures that serve as both mechanical linkages and centers of intracellular signal transduction [22-24]. They assemble as nascent adhesions, mature into focal complexes, focal adhesions and fibrillar adhesions and disassemble [23]. Consequently, FAs exhibit different morphological maturation states throughout their lifetime and this is thought to regulate their behavior. For example, small, nascent FAs, transmit strong forces and serve as traction points for propulsive forces to move the cell body forward [25,26]. They also generate signals for protrusion by activating actin accessory proteins [27-31]. Under tension, these small FAs can mature into larger focal complexes, focal adhesions and fibrillar complexes with different force transmission characteristics and propensities for protrusion signaling [32-34]. Several morphological characteristics have been used to predict traction force and cell migration speed including FA protein density, number per cell, sliding speed, lifetime, size and elongation [22,23,25,33-35]. These morphological characteristics have begun to be quantitatively measured [36,37] and the distributions properly quantified [38]. However, their direct correlation to migratory states as well as their response to extracellular cues like EGF is unknown.

Protrusion is mediated by actin polymerization, whereas retraction is driven through myosin II activity and actin depolymerization [39,40]. Protrusion and retraction can either occur continuously in spatially confined regions as in keratocyte migration or it can occur in cycles or waves of protrusion that move laterally along the edge [41-43]. This has been characterized in several cell types when cells are either spreading [44] or migrating [41-43]. In fact a recent paper has shown that slower migrating keratocytes employ lateral protrusion waves [43]. While the timing of the cycles and the propagation of the waves is dependent on intracellular pathways, very little work has been done to examine how protrusion is quantitatively altered in response to extracellular stimuli like EGF.

To understand the relationship between EGF-stimulated cell migration, FA properties and protrusion dynamics, we imaged metastatic (MTLn3) and non-metastatic (MTC) cell lines. We analyzed the cell migration speed and persistence under various EGF stimulation conditions and found that EGF moderately increased the median migration rate and persistence of MTLn3 cells, whereas it had no significant effect on the speed and persistence of MTC cells. Interestingly, higher concentrations of EGF broadened the distributions and increased the coefficient of variation of both the migration rate and persistence of MTLn3 cells, but not MTC cells. When data was binned based on EGF stimulation conditions, FA intensity and FA number per cell showed the largest difference among stimulation groups. FA intensity decreased with increasing EGF concentration and FA number per cell was highest under intermediate stimulation conditions. No difference in protrusion behavior was observed. However, when data was binned based on cell migration speed, FA intensity and not FA number per cell showed the largest difference among groups. FA intensity was lower for fast migrating cells. Additionally, waves of protrusion tended to correlate with fast migrating cells. Consequently, low FA intensity and waves of protrusion are markers for fast migrating cells, but these characteristics are only partially predictive of EGF stimulation conditions because of the large cell-to-cell variability in response to EGF.

## Results

### EGF stimulation broadens the distributions of migration speed and persistence of MTLn3 cells

**Figure 1:**
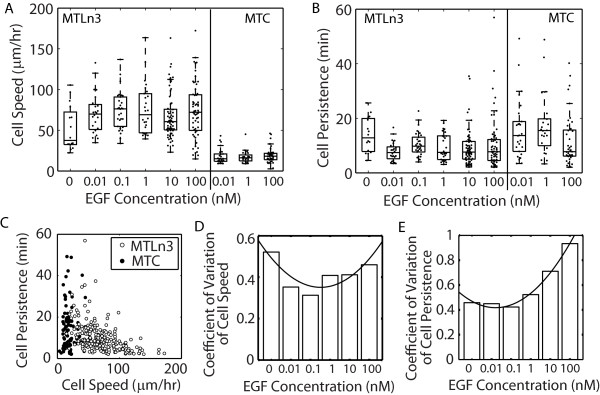
**Increasing EGF broadens the distribution of both cell speed and persistence of MTLn3 cells while not changing MTC cell migration. A**. cell speed and (um/hr) in the axis description overlaps. **B**. Cell persistence of MTLn3 (left) and MTC (right) under different EGF concentrations. Data for individual cells are shown as black dots. On each box, the central marker is the median; the edges of the box are the 25^th^ and 75^th^ percentiles; the whiskers extend to the most extreme data points not considered outliers. (For MTLn3, *N*_*0*_ = 16, *N*_*0.01*_ = 24, *N*_*0.1*_ = 30, *N*_*1*_ = 26, *N*_*10*_ = 65, *N*_*100*_ = 60; For MTC, *N*_*0.01*_ = 26, *N*_*1*_ = 24, *N*_*100*_ = 41.) **C**. Correlation between cell speed and persistence for MTLn3 cells (white circles, *N* = 221) and MTC cells (black circles, *N* = 91). **D**. Coefficient of variation of cell speed for MTLn3 cells as a function of EGF concentration. **E**. Coefficient of variation of cell persistence for MTLn3 cells as a function of EGF concentration. Curves were fitted to a 2^nd^ order polynomial and meant only to guide the eyes.

In many cell types EGF has been reported to enhance the mean migration speed. However, the long term migration response of individual cells after challenge with EGF in this model system is not known. Consequently, we examined cell migration under various doses of EGF in both adenocarcinoma cells (MTLn3) and non-metastatic cells taken from the same tumor (MTC) (Figure 1). *In vivo*, EGF concentrations in serum can be between 0.1-2 nM, with local tissue concentrations as high as ~20 nM [13]. Consequently, MTLn3 and MTC cell lines were stimulated with a wide range of EGF concentrations (0–100 nM). Cell tracks were generated (Additional file 1: Figure S1) and cell speed and persistence were calculated based on fitting the mean squared displacement with a random walk model [45,46]. Contrary to previous reports in other cell lines, EGF stimulation only increased the median speed and decreased the median persistence slightly in MTLn3 cells and acted more like an on-off switch between no EGF stimulation and EGF stimulation (Figure 1A and B). In addition, there was no dose response in either median speed or persistence of MTC cells (Figure 1A and B). Cell persistence decreased somewhat with increasing cell speed in MTLn3 cells, but MTC showed no such correlation, populating a much lower range of migration speeds (Figure 1C) with roughly the same range of persistence times. Interestingly, at higher EGF concentrations, MTLn3 cells both migrated faster and slower than those at lower EGF concentrations (Figure 1A). On the other hand, MTC cells did not show this same behavior. The coefficient of variation (standard deviation/mean) increased in a dose dependent manner for both cell speed and persistence in MTLn3 cells (Figure 1 D and E).

Given that EGF did not dramatically affect the median migration, we grouped cells according to no (0 nM EGF), low (0.01 and 0.1 nM) and high (1, 10 and 100 nM) EGF stimulation. Additionally, since the variability in cell migration speed seems to be an additional feature of the data, we also grouped cells based on cell migration speed. A *k*-means clustering algorithm for a cluster number equal to two was applied to the migration speeds of all cells under different EGF concentrations. The cutoff speed between slow and fast migration cells was found to be 42 μm/hr. Thus we assigned cells with speeds of greater than 42 μm/hr to the fast migrating group and cells with speeds of less than 42 μm/hr to the slow migrating group. Having grouped cells in two different ways, we wanted to examine FA characteristics and protrusion dynamics to see if certain signatures were exhibited by EGF stimulated cells or fast moving cells.

### The distribution of FA characteristics differ under different EGF stimulation conditions and between fast and slow migrating cells

**Figure 2:**
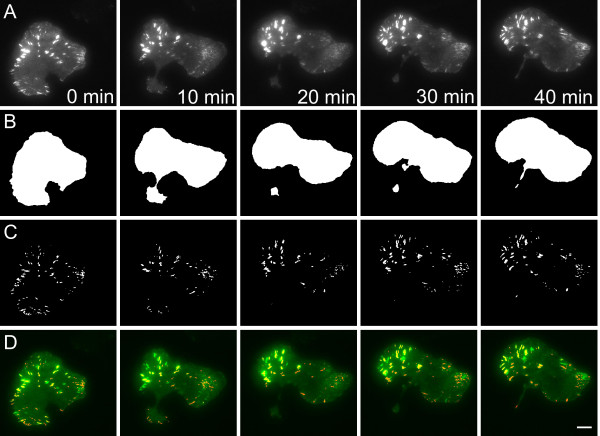
**Time-lapse series of FA dynamics in MTLn3 cells.** MTLn3 cell expressing paxillin-EGFP and stimulated with 0.01 nM EGF is shown. Images were taken at 0, 10, 20, 30 and 40 min. **A**. Original total internal reflection fluorescence (TIRF) images of cells with FAs. **B**. Binary images of whole cell masks after segmentation. **C**. Binary images of FA masks after segmentation. **D**. Composite images of tracked FAs within the cell, where green represents original images and red represents the segmented FA masks. The scale bar is 10 μm.

In order to measure the FA characteristics of FA intensity, number per cell, size, speed, lifetime and elongation, we used TIRF microscopy to observe FAs within a single cell transfected with paxillin-EGFP, a component that marks FAs throughout their entire lifetime. Expression of paxillin-EGFP did not seem to alter the migration speed, nor was the expression dependent on EGF stimulation (Additional file 2: Figure S2). In MTLn3 cells, many FAs assembled, matured and disassembled over several minutes, so images were taken every 10s for 40–60 minutes (Figure 2A), with little influence of photobleaching (Additional file 3: Figure S3). A segmentation and tracking algorithm was used to quantify FA characteristics (Figure 2B-D) and time-resolved data of focal adhesion characteristics ( Additional file 4: Figure S4) [37]. We categorized by eye the FA tracking results of different EGF concentrations and scored them as poor, good and excellent. The example shown in Figure 2 was scored as good. Most tracks at each EGF condition resulted in more than 70 % that were either good or excellent (Table 1).

**Table 1:** Qualitative assessment of tracking results

**EGF (nM)**	**CELL NUMBER**	**POOR**	**GOOD**	**EXCELLENT**
0	5	0	60 %	40 %
0.01	10	20 %	70 %	10 %
0.1	10	50 %	20 %	30 %
1	10	10 %	20 %	70 %
10	12	17 %	33 %	50 %
100	8	25 %	50 %	25 %
Total	55	22 %	40 %	38 %

Percentages of poor, good or excellent FA tracking results under different EGF concentrations are shown. An example of a cell that was rated as good is shown in Figure 2.

**Figure 3:**
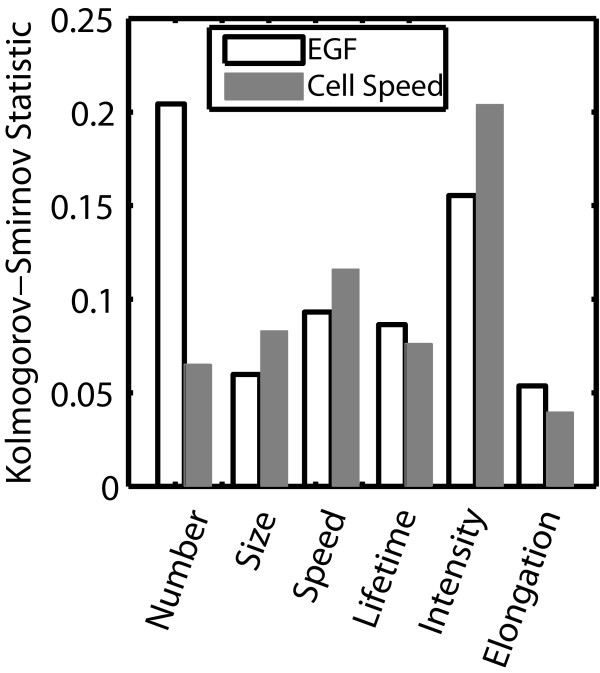
**Quantification of the difference between experimental distributions of FA properties.** Cells were binned based on EGF concentration (no (0 nM), low (0.1 and 0.01 nM) and high (100, 10 and 1 nM)) or cell speed (low (<42 μm/hr) or high (>42 μm/hr)). The Kolmogorov-Smirnov statistic was the average of three pair-wise comparisons (EGF) or simply the pair-wise comparison (cell speed). A larger value for the Kolmogorov-Smirnov statistic signifies a higher probability that there are differences between groups.

**Figure 4:**
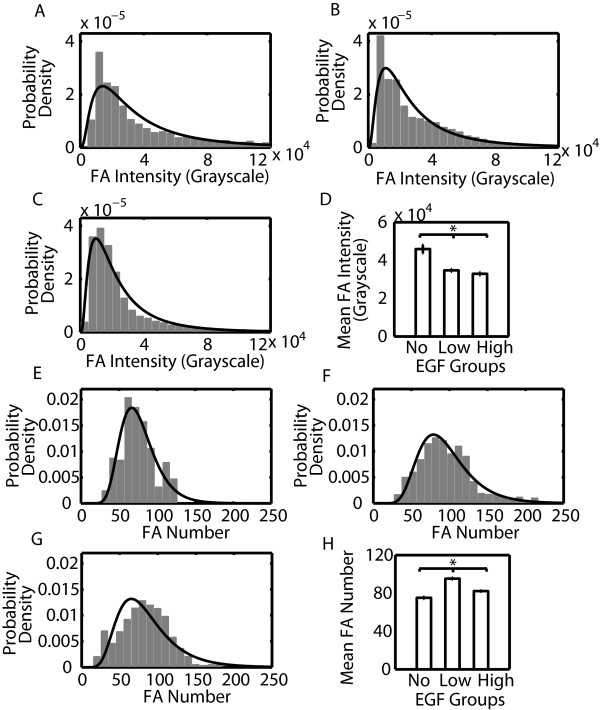
**FA intensity decreases with increasing EGF concentration and number per cell is maximal at intermediate EGF concentrations.** Histograms of FA intensity A.-C. and number per cell E.-G. were generated by dividing cells into three EGF stimulation groups (A., E. no EGF (0 nM), B., F. low EGF (0.01 and 0.1 nM) and C., G. high EGF (1, 10 and 100 nM)). Histograms were fitted with lognormal probability distributions. The mean values of FA D. intensity and H. number per cell are also shown. The number of measurements of FA number per cell is the product of the average number of frames and the cell number. The number of measurements of FA properties is the product of the average FA number and the cell number. Intensity: *N*_*cell,no*_ = 5, *N*_*FA,no*_ = 1963, *N*_*cell,low*_ = 20, *N*_*FA,low*_ = 13,024, *N*_*cell,high*_ = 30, *N*_*FA,high*_ = 14,648. Number per cell: *N*_*cell,no*_ = 5, *N*_*FA,no*_ = 1545, *N*_*cell,low*_ = 20, *N*_*FA,low*_ = 5909, *N*_*cell,high*_ = 30, *N*_*FA,high*_ = 8937. Error bars are 95 % confidence intervals and asterisks denote *p* < 0.01.

**Figure 5:**
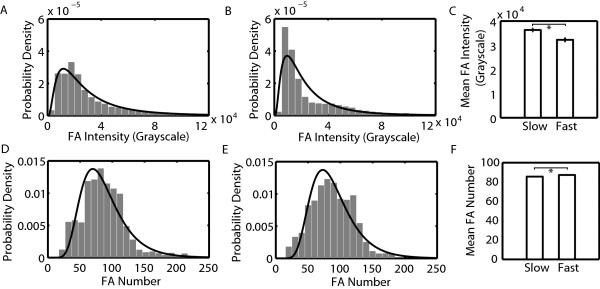
**FA intensity is lower in fast migrating cells and FA number per cell is slightly higher in fast migrating cells.** Histograms of FA intensity A.-B. and FA number per cell D.-E. were generated by dividing cells into two cell migration speed groups (A., D. slow (<42 μm/hr) and B., E. fast (>42 μm/hr)). Histograms were fit with lognormal probability distributions. The mean values of FA C. intensity and F. number per cell are also shown. The number of measurements of FA number per cell is the product of the average number of frames and the cell number. The number of measurements of FA properties is the product of the average FA number and the cell number. Intensity: *N*_*cell,slow*_ = 21, *N*_*FA,slow*_ = 12,773, *N*_*cell,fast*_ = 34, *N*_*FA,fast*_ = 16,862. Number per cell: *N*_*cell,slow*_ = 21, *N*_*FA,slow*_ = 6146, *N*_*cell,fast*_ = 34, *N*_*FA,fast*_ = 10,245. Error bars are 95 % confidence intervals and asterisks denote *p* < 0.01.

FA characteristics can be ordered based on the magnitude in the difference between either the no, low and high EGF stimulation conditions or between slow and fast migrating cells. This magnitude was quantified by the Kolmogorov-Smirnov statistic (Figure 3). When this statistic is large, it is more likely that there is a difference in distributions between groups. FA intensity and number per cell showed the largest values, so we decided to focus on these characteristics. Distributions of all other FA characteristics are shown as Additional file 5: Figure S5 and Additional file 6: Figure S6 and a summary of relevant ranges of these FA characteristics under different conditions is shown in Table 2. Most FA characteristics fit best to either lognormal or Weibull probability distribution functions. As EGF concentration increased from no to low to high, FA intensity decreased (Figure 4). FA number per cell on the other hand showed highest numbers at low concentrations of EGF. Both FA intensity and number per cell showed strong differences between EGF concentration groups (Figure 3). When cells were grouped based on migration speed, FA intensity was lower for fast migrating cells (Figure 5). However, distributions for FA number per cell were now much less, indicating that this characteristic shows poorer correlation with migration speed (Figure 3). Having identified some FA characteristics that correlate with either EGF stimulation conditions or cell speed, we were interested if any protrusion characteristics showed difference among groups.

**Table 2:** Summary of FA characteristics in fast migrating cells and those stimulated with low and high EGF concentrations

**Properties**	**Fast Migrating Cells**	**Low EGF**	**High EGF**
FA Number	> 110	> 86	> 97
FA Size (μm^2^)	0.30 - 3.0	0.18 - 3.0	0.24 – 3.0
FA Sliding Speed (μm/hr)	> 12	> 12	> 12
FA Lifetime (s)	0 - 440	160 - 530	160 - 620
FA Intensity (grayscale)	< 22,000	<25,000	<25,000
FA Elongation	N/A	1.3 – 2.1	1.3 – 2.2

The ranges indicates areas of higher probability of fast migrating cells compared to slow migrating cells, cells stimulated with low concentrations of EGF compared to those exposed to no EGF and cells stimulated with high concentrations of EGF compared to those exposed to no EGF. The ranges were determined by calculating crossover points between the fit distributions. N/A means no crossover points.

### Unique spatial organization of protrusion and retraction is exhibited in fast migrating cells

**Figure 6:**
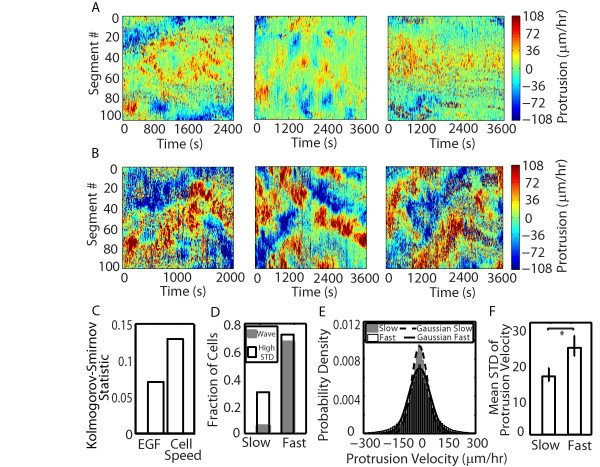
**The spatial control of protrusion differs between slow and fast migrating cells. A**. Protrusion velocity map for slow migrating cells at 0.01, 1, and 100 nM EGF from left to right. **B**. Protrusion velocity map for fast migrating cells at 0.01, 1, and 100 nM EGF from left to right. The cell edge was divided into 100 segments and the average protrusion rate in each segment was determined over time. Red represents fast protrusion, green represents quiescence and blue represents fast retraction. **C**. The Kolmogorov-Smirnov statistic was the average of three pair-wise comparisons (EGF) or simply the pair-wise comparison (cell speed). A larger value for the Kolmogorov-Smirnov statistic signifies a higher probability that there are differences between groups. **D**. The fraction of cells with lateral waves (gray bars) or high standard deviation (STD) of protrusion velocity (white bars) between slow and fast migrating cells. **E**. Histograms of protrusion/retraction velocity between slow migrating cells (gray bars) and fast migrating cells (white bars). Histograms of slow (dot lines) and fast migrating cells (solid lines) were fit with Gaussian distribution. **F**. The mean values of standard deviation (STD) of protrusion velocity between slow and fast migrating cells were also shown. *N*_*slow*_ = 34, *N*_*fast*_ = 24. Error bars are 95 % confidence intervals and asterisks denote *p* < 0.01.

We assessed differences in protrusion and retraction behavior under different EGF stimulation conditions and between the slow and fast migrating cells. The cell-average protrusion and retraction velocities would be faster in the fast migrating cells resulting in the increased migration rate; however different patterns of protrusion could lead to the same average value, so we analyzed local protrusion behavior. One prominent feature that we observed was traveling waves of protrusion along the edge of the cell. This traveling wave behavior had the effect of broadening of the protrusion velocity distribution. Upon qualitative examination, traveling waves did not seem to be linked to EGF stimulation conditions. Additionally, slow migrating cells usually showed large quiescent areas (green) and random, disorganized protrusion and retraction behavior (Figure 6A). Only fast migrating cells showed traveling waves of protrusion (Figure 6B). We examined the difference in protrusion velocity distributions in the same way that we examined distributions of FA properties (Figure 3). The Kolmogorov-Smirnov statistic was smaller when comparing EGF stimulation conditions than it was when comparing cell migration speeds (Figure 6C). This suggested that waves as described by a wide protrusion velocity distribution correlate with differences in migration speed and not EGF concentration. We computed the fraction of cells with waves and measured the standard deviation of the protrusion velocity distribution in slow and fast migrating cells. Both waves and high standard deviations were features of fast migrating cells (Figure 6D, E and F). Consequently, fast cells tend to organize their protrusion in a qualitatively different way than slow migrating cells and this does not necessarily correlate with EGF stimulation.

## Discussion

Variability in cell response to environmental cues is becoming a more appreciated phenomenon that can drive how populations of cells respond to their environment. Cell-to-cell variability can arise from heterogeneity in protein level [47,48] or organization of cellular structures such as the membrane [49] or the cytoskeleton [50]. Interestingly, this variability can be enhanced by extracellular stimuli [51]. The idea that variability can be enhanced under certain conditions sets up the interesting possibility that the mean response is a relatively poor statistical metric. Rather, the distribution itself or the standard deviation or another parameter that characterizes the distribution may be more appropriate. The obvious result of this dependence on the distribution is a sensitizing of a subpopulation of cells to particular environments. This is acutely evident in pathologies such as cancer metastasis, where subpopulations of cells are selected based on differing responses to the tumor microenvironment. Therefore, the fastest cells most likely drive metastasis, whereas the average cell migration rate might be less important. We showed that the distribution of cell migration speed and persistence is very much regulated under EGF stimulation, even though the average response differs marginally. Indeed, this has been demonstrated previously. [18] Ware et al. generated distributions of migration rate in response to no EGF or high EGF concentration. However, the focus of that paper was primarily on the changes in the average migration response and the widening of the distribution in response to EGF was evident, but not discussed. What causes this widening? Heterogeneity in the local ECM concentration might play a role. We have examined collagen coverage and it tends to be fairly homogeneous at the resolution of the light microscope (~100 nm) and we observed cells in close proximity that varied greatly with respect to their migration speed. However, ECM inhomogeneity cannot be fully dismissed as a possible cause for the cell-to-cell variability. Another cause of the cell-to-cell variability might be autocrine or paracrine signaling. MTLn3 cells are known to secrete other EGF receptor ligands, namely TGF-α [52]. However, we did not observe clustering of migration speeds around sources. Often cells in the same clusters showed distinct behavior. A third possibility is that concentrations of signaling, adhesion or cytoskeletal regulatory proteins might contribute to the heterogeneity. This might be the most probable cause of the cell-to-cell variability; however determining which specific components might contribute to this is the subject of further investigation.

**Figure 7:**
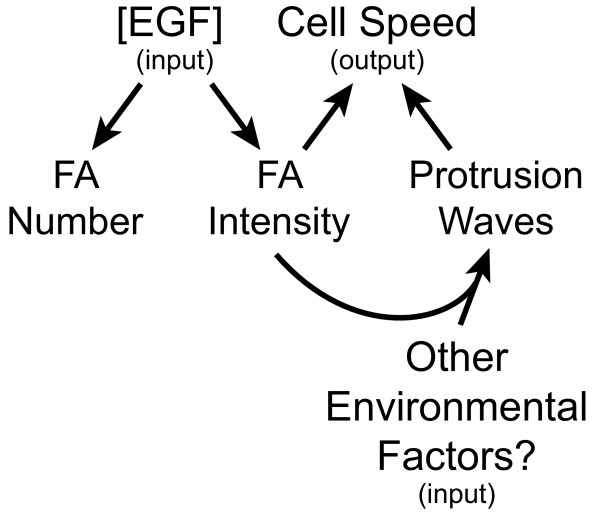
**Different adhesion and protrusion characteristics correlate with EGF stimulation and cell speed.** Cells can either be grouped based on EGF concentration or cell speed. EGF concentration is considered an input that acts to regulate adhesion and protrusion characteristics, whereas cell speed is an output that acts to integrate information determined by inputs such as EGF concentration. Both FA intensity and number per cell correlate with EGF concentration, whereas FA intensity and the presence or absence of protrusion waves correlate with cell speed. Cell speed could be regulated by EGF through changes in FA intensity, but other inputs are most likely needed to regulate the presence of protrusion waves, since EGF concentration correlates poorly with the presence or absence of protrusion waves.

EGF does seem to regulate some FA characteristics, namely FA intensity and number per cell. FA intensity decreases as EGF stimulation increases. FA number per cell is highest at low EGF concentrations, suggesting that either the assembly is maximized or disassembly is minimized at this point. EGF is known to alter actin cytoskeleton dynamics, perhaps resulting in enhanced assembly dynamics. Alternatively, EGF is also known to upregulate calpain, a protease involved in disassembly, which might be largest at high EGF concentrations. Interestingly, FA number per cell does not seem to affect cell migration rate. Rather, low FA intensity seems to correlate with fast migrating cells. This might be a direct link between EGF stimulation and cell migration speed regulation (Figure 7). Zaidel-Bar et al. observed that the localization of paxillin in large FAs did not affect the rate of protrusion of the nearby lamella. However, paxillin association with focal complexes was inversely correlated with the rate of local protrusion. Thus, focal complexes containing relatively low levels of paxillin were found in fast protrusions [29]. While seemingly less important as measured by the Kolmogorov-Smirnov statistic, we also found that fast migrating cells contain FAs with intermediate sizes and FAs with intermediate sliding speeds and FAs with short lifetimes (Table 2 and Additional file 6: Figure S6). The fact that fast migrating requires intermediate sized FAs (0.3-3 μm^2^) is not surprising. Focal complexes, small FAs are traditionally thought to occupy this range of areas [24]. These smaller FAs are usually located near the leading edge and transmit strong propulsive traction forces needed during fast migration. Larger, mature FAs exert weaker forces [25] and “supermature” fibrillar adhesions [53] are involved in ECM remodeling, both processes that are typically seen in slower migrating cells. Fast migrating cells also contained FAs with intermediate speed. FA speed affects cell speed in complicated ways due to its spatial regulation. For example, Smilenov et al. found that fibroblasts with stationary FAs tend to transmit large forces and result in migratory cells [35]. However, Diener et al. found that FAs moved with a sliding speed of 4 μm/hr in migrating human osteosarcoma cells on collagen-coated coverslips [54] and FAs at the trailing edge are pulled forward at rates of >5-10 μm/hr [55]. Lifetime was minimal in fast migrating cells. Others have shown that FAs with short lifetimes correlate with fast migrating cells, in line with what we observe [28].

Given that local protrusion is linked to FA intensity and that FA intensity was lowest in fast migrating cells we were encouraged to examine the protrusion dynamics under different stimulation conditions. These cells are known to respond acutely to EGF stimulation with two peaks of barbed end formation resulting in a robust protrusion response [56]. However, cells are often not exposed to these acute signals *in vivo* and so we asked how protrusion changes under chronic EGF stimulation. We found that while EGF stimulation condition correlated poorly with lateral waves generated in cells, fast migrating cells usually generated lateral waves of protrusion as has been seen elsewhere [41-43,57].The existence of lateral protrusion waves suggests locally activated feedback loops that travel laterally along the edge of the cell [43,58]. This positive feedback loop operates through adhesion signaling for protrusion and protrusion resulting in more adhesions [59]. How does this behavior relate to migration rate? Barnhart et al. noticed that keratocytes migrating on more adhesive substrates generated these lateral waves and migrated with a slower speed [43]. We see an opposite relationship, where high speeds result in lateral waves of protrusion. This difference may be related to the differences in cytoskeletal organization and in fact morphology between these cells. Keratocytes adopt highly regular persistent cytoskeletal structure and cellular morphology resulting in extremely fast migration speeds (500–600 μm/hr). MTLn3 cells on the other hand have a varied cytoskeletal structure and cellular morphology and are much slower (<100 μm/hr). Consequently, highly organized, persistent protrusion that is seen in keratocytes results in the fastest migrating cells. Less efficient, but somewhat organized lateral protrusion seen in both keratocytes and MTLn3 cells results in intermediate speeds. Poorly organized protrusion seen in MTLn3 cells results in slow speeds. Local differences in ECM in our system might explain why EGF is not a primary driver for fast migrating cells or lateral protrusion waves, leading high cell-to-cell variability (Figure 7).

## Conclusions

EGF was found to broaden the distribution of cell migration rates, generating both faster and slower cells, but not dramatically affecting the average response. Several different adhesion and protrusion characteristics correlated with EGF stimulation and cell migration speed, however there is a hierarchy of these correlations. FA intensity and number per cell correlate with EGF stimulation conditions. FA intensity decreases with increasing EGF stimulation and FA number per cell is highest at low EGF stimulation conditions. In contrast, FA intensity and not number per cell as well as protrusion waves correlate with cell speed. Fast cells are marked by low FA intensity and protrusion waves. Consequently, while EGF stimulation could regulate FA intensity to modulate cell speed directly or by partially activating protrusion waves, other environmental factors most likely lead to protrusion waves. Adhesion and protrusion characteristics that do not correlate with EGF stimulation condition but do correlate with cell migration speed constitute the most sensitive outputs for identifying why cells respond differently to EGF. The idea that EGF can both increase and decrease the migration speed of individual cells in a population has particular relevance to cancer metastasis where the microenvironment can select subpopulations based on some adhesion and protrusion characteristics, leading to a more invasive phenotype as would be seen if all cells responded like an “average” cell.

## Methods

### Materials

Cell culture media was α-MEM medium with L-glutamine (Invitrogen) containing 5 % fetal bovine serum (Invitrogen) and 1 % penicillin-streptomycin (Invitrogen). Collagen and poly-L-lysine (PLL) solution contained 3 μg/ml of rat tail collagen I (Invitrogen) and 2 μg/ml of poly-L-lysine hydrochloride (Sigma), dissolved in 0.5 M acetic acid (Fisher) and sterilized under ultraviolet light for 30 minutes. Serum free imaging media was α-MEM medium without phenol red (Invitrogen) containing 1 mg/ml bovine serum albumin (Sigma), 12 mM HEPES (Fisher), and 1 % penicillin-streptomycin (Invitrogen), adjusted to pH 7.4 and filtered through 0.22 μm pore size filter (Millipore, Fisher).

### Cell culture

Rat mammary adenocarcinoma cell lines (metastatic MTLn3 and non-metastatic MTC) were obtained from Dr. Jeffrey E. Segall (Albert Einstein college of Medicine). Cell lines were derived from the 13762NF rat mammary adenocarcinoma tumor. Cells were maintained in cell culture media at 37 °C in 5 % CO_2_ and were passed every 2 or 3 days. Collagen and PLL solution was incubated on 22 × 22 mm squeaky cleaned coverslips (Corning, Fisher) at room temperature for 1 hour. Cells were seeded on coverslips with collagen and PLL and incubated for 24 ~ 48 hours at 37 °C in 5 % CO_2_ (50,000 ~ 100,000 cells/coverslip).

### Cell migration assay

MTLn3 and MTC cells were incubated on coverslips with collagen and PLL for 48 hours and were switched to serum free imaging media for 2 hours. Coverslips were mounted onto glass slide chambers in serum free imaging media with different concentrations of EGF (0, 0.01, 0.1, 1, 10 and 100nM EGF). Chambers were maintained at 37 °C for 2 hours and then imaged on a heated stage every 2 minutes for 8 hours. Phase contrast time-lapse images were captured at 20× (NA 0.50, Nikon) with a charge-coupled device (CoolSNAP HQ2, Photometrics) attached to an inverted microscope (Eclipse Ti, Nikon). Cell centroids were identified and tracked manually by MTrackJ plugins of ImageJ. Single cell speed, *S*, and directional persistence time, *P*, were obtained by fitting these to the persistent random walk equation [45,46]:

(1) $$\langle d^{2}\left(t\right)\rangle =2S^{2}P\left[t-P\left(1-e^{-t/P}\right)\right]$$

using a non-linear least squares regression analysis. The sampling time is every two minutes for 6–8 hours. The mean-squared displacement was constructed using non-overlapping time intervals. Consequently, the model was fitted to data up to a 30 min time lag due to the small number of displacements (<12-16) at time lags greater than 30 min. To quantify protrusion rate we used a constrained optimization program to measure the protrusion and retraction rates from masked images as done previously [42]. The cell edge was segmented into 100 sectors. The average protrusion rate in these sectors was calculated over time.

### Fluorecence imaging

MTLn3 cells were incubated on coverslips with collagen and PLL for 24 hours and transfected with paxillin-EGFP and Fugene 6 (Roche) according to the manufacturer’s protocol (6 μl of Fugene 6 and 3 μg of EGFP-paxillin). After one hour transfection, the media was changed to cell culture media and the transfected cells were maintained at 37 °C in 5 % CO_2_ for 23 hours. Then the cells were switched to serum free imaging media for 2 hours. Coverslips were mounted onto glass slide chambers in serum free imaging media with different concentrations of EGF (0, 0.01, 0.1, 1, 10 and 100 nM EGF). Chambers were maintained at 37 °C for 2 hours and then imaged on a heated stage every 10 seconds for 40 ~ 60 minutes. TIRF images were captured at 60× oil objective (NA 1.49, Nikon) equipped with a TIRF illuminator and fiber optic-coupled laser illumination. The 488 nm laser line of an air-cooled tunable Argon laser (Omnichrome Model 543-AP-A01, Melles Griot) was reflected off a dichroic mirror (89000 ET-QUAD, Chroma). Camera and shutter were controlled by μManager 1.3. An automated segmentation and tracking algorithm was utilized for large-scale analysis of FA dynamics [37]. FAs smaller than 0.05 μm^2^ and larger than 10 μm^2^ were excluded from our analysis because they represent either FAs consisting of less than three pixels or several FAs clustered together. FA fluorescence intensities were calibrated to the standard condition of 1 mW laser power with a 300 ms exposure time, so FA intensity should be directly proportional to protein level across all samples.

### Statistical analysis

All graphs and statistical analyses were done using JMP and Matlab software. Distributions of FA properties were constructed in the following ways. FA number as described in the results section is more precisely a FA number per cell and consequently the distribution was generated by using the calculated FA number per cell at each time point during the experiment for each cell. Consequently, the number of measurements of FA number per cell is the product of the average number of frames and the cell number. All the other distributions of FA properties were generated by using the time-averaged FA property for each FA in each cell. Consequently, the number of measurements of FA properties is the product of the average FA number and the cell number. Differences between conditions under various EGF concentrations and cell migration speeds were quantified by calculating the Kolmogorov-Smirnov statistic using the Matlab function . Model distributions were fitted by minimizing the Kolmogorov-Smirnov statistic using the Matlab function between the experimental distribution and the model distribution. To determine the statistical differences of the mean values between conditions under various EGF concentrations and cell migration speeds, a student’s t-test was utilized and a *p* < 0.01 was deemed significant.

## Competing interests

The authors declare that they have no competing interests.

## Authors’ contributions

YH carried out the bulk of the experiments. SH conducted the long term migration experiments. YH and ICS analyzed the results and wrote the paper. All authors read and approved the final manuscript.

## Supplementary Material

Additional file 1 Figure S1.Migration trajectories of typical cells under different EGF concentrations. Three cell tracks were chosen randomly under each EGF concentrations and labeled with different colors. All trajectories were aligned to the starting point (0, 0). Click here for file

Additional file 2 Figure S2.Paxillin-EGFP expression levels for cells with different cell speeds and under different EGF stimulation conditions. Mean intensity of individual cells as a function of A. cell speed, and B. EGF concentrations. *N*_*cell*_ = 56. Click here for file

Additional file 3 Figure S3.Average paxillin-EGFP intensity in cells as a function of time. A. Mean FA intensity and B. mean intensity of the whole cell for all cells as a function of time. Mean FA Intensity: *N*_*cells*_ = 55. Mean Intensity *N*_*cells*_ = 53. Click here for file

Additional file 4 **Time-resolved data of mean FA intensity and FA number per cell. A.** Mean FA intensity and C. FA number per cell for the cell shown in Figure 2 under 0.01 nM EGF stimulation as a function of time. B. FA mean intensity and D. number per cell of five typical cells under different EGF concentrations as a function of time. Click here for file

Additional file 5 Figure S5.Distributions of FA size, speed, lifetime and elongation for different EGF stimulation conditions. Histograms of FA size A.-C., speed E.-G., lifetime I.-K. and size M.-O. were generated by dividing cells into three EGF stimulation groups (A., E., I. and M., no EGF (0 nM), B., F., J. and N. low EGF (0.01 and 0.1 nM) and C., G.,K. and O. high EGF (1, 10 and 100 nM)). Histograms were fitted with lognormal probability distributions except for speed, which was fitted with a Weibull probability distribution. The mean values of FA D. size, H. speed, L. lifetime and P. elongation are also shown. The number of measurements of FA properties is the product of the average FA number and the cell number. Size, speed, lifetime and elongation: *N*_*cell,no*_ = 5, *N*_*FA,no*_ = 1963, *N*_*cell,low*_ = 20, *N*_*FA,low*_ = 13,024, *N*_*cell,high*_ = 30, *N*_*FA,high*_ = 14,648. Error bars are 95 % confidence intervals and asterisks denote *p* < 0.01. Click here for file

Additional file 6 Figure S6.Distributions of FA size, speed, lifetime and elongation for slow and fast migrating cells. Histograms of FA size A.-B., speed D.-E., lifetime G.-H. and size J.-K. were generated by dividing cells into slow and fast migrating groups (A., D., G. and J. slow migrating cells and B., E., H. and K. fast migrating cells). Histograms were fitted with lognormal probability distributions except for speed, which was fitted with a Weibull probability distribution. The mean values of FA C. size, F. speed, I. lifetime and L. elongation are also shown. The number of measurements of FA properties is the product of the average FA number and the cell number. Size, speed, lifetime and elongation: *N*_*cell,slow*_ = 21, *N*_*FA,slow*_ = 12,773, *N*_*cell,fast*_ = 34, *N*_*FA,fast*_ = 16,842. Error bars are 95 % confidence intervals and asterisks denote *p* < 0.01. Click here for file
